# Urinary Concentrations of 2,4-Dichlorophenol and 2,5-Dichlorophenol in the U.S. Population (National Health and Nutrition Examination Survey, 2003–2010): Trends and Predictors

**DOI:** 10.1289/ehp.1306816

**Published:** 2014-01-22

**Authors:** Xiaoyun Ye, Lee-Yang Wong, Xiaoliu Zhou, Antonia M. Calafat

**Affiliations:** Division of Laboratory Sciences, National Center for Environmental Health, Centers for Disease Control and Prevention, Atlanta, Georgia, USA

## Abstract

Background: 2,4-Dichlorophenol (2,4-DCP), 2,5-dichlorophenol (2,5-DCP), and their precursors are widely used in industry and in consumer products. Urinary concentrations of these dichlorophenols (DCPs) have been measured as part of four National Health and Nutrition Examination Survey (NHANES) cycles in order to assess the exposure to these compounds or their precursors among the general U.S. population.

Objectives: We identified predictors and evaluated trends in DCP concentrations according to race/ethnicity, age, sex, family income, and housing type.

Methods: We used analysis of covariance to examine associations of various demographic parameters and survey cycle with urinary concentrations of DCPs during NHANES 2003–2010. We also conducted weighted logistic regressions to estimate associations of DCP concentrations above the 95th percentile with housing type, race/ethnicity, and income.

Results: We detected DCPs in at least 81% of participants. Geometric mean (GM) urinary concentrations were higher for 2,5-DCP (6.1–12.9 μg/L) than 2,4-DCP (0.8–1.0 μg/L) throughout 2003–2010. Adjusted GM concentrations of the DCPs among children (6–11 years of age) and adults > 60 years of age were higher than among adolescents and other adults. Adjusted GM concentrations among non-Hispanic whites were lower than among non-Hispanic blacks and Mexican Americans, although differences according to race/ethnicity were less pronounced among participants in high-income households. Among non-Hispanic blacks and Mexican Americans, adjusted GM concentrations were lowest among high-income participants relative to other income groups, with a monotonic decrease with increasing income among Mexican Americans. Type of housing and race/ethnicity were significant predictors of DCP urinary concentrations above the 95th percentile. Furthermore, urinary DCP concentrations have showed a downward trend since 2003.

Conclusions: Exposure to DCPs and their precursors was prevalent in the general U.S. population in 2003–2010. We identified age and race/ethnicity, family income, and housing type as predictors of exposure to these compounds.

Citation: Ye X, Wong LY, Zhou X, Calafat AM. 2014. Urinary concentrations of 2,4-dichlorophenol and 2,5-dichlorophenol in the U.S. population (National Health and Nutrition Examination Survey, 2003–2010): trends and predictors. Environ Health Perspect 122:351–355; http://dx.doi.org/10.1289/ehp.1306816

## Introduction

2,5-Dichlorophenol (2,5-DCP) is a major metabolite of 1,4-dichlorobenzene (1,4-D), which has been used as a chemical intermediate for the manufacture of dyes and pharmaceutical and agricultural products, as a moth repellent, and as a space deodorant for industrial and indoor home applications [Hazardous Substances Data Bank (HSDB) 2014; International Programme on Chemical Safety (IPCS) 1989]. 2,4-Dichlorophenol (2,4-DCP) is primarily used in the production of phenoxy acid herbicides such as 2,4-diphenoxyacetic acid (2,4-D) and for the synthesis of pharmaceuticals and antiseptics (HSDB 2014). 2,4-DCP can also enter the environment as a degradation product of triclosan, an antimicrobial agent (Canosa et al. 2005), and both 2,4-DCP and 2,5-DCP are by-products of the chlorination of municipal drinking water and industrial waste water (HSDB 2014). The U.S. Environmental Protection Agency (EPA 2002) considers dichlorophenols (DCPs) to be hazardous pollutants. General population exposure to DCPs and their precursors can occur through industrial and indoor air pollution, diet, and the use of pesticides and consumer products (HSDB 2014).

Chlorinated phenols are toxic for a wide range of wildlife organisms and humans (Hsiao et al. 2009; IPCS 1989; Takahashi et al. 2011). In 1987, the International Agency for Research on Cancer (IARC) classified 1,4-D as a carcinogen in animals [IARC 1999; National Toxicology Program (NTP) 2011]. The Agency for Toxic Substances and Disease Registry (ATSDR) and IARC have classified 1,4-D as a suspected human carcinogen (ATSDR 2006; IARC 1999). Because of the potential adverse health effects upon exposure to these DCPs or their precursors (Buttke et al. 2012; Chevrier et al. 2012; Hsiao et al. 2009; Jerschow et al. 2012; Philippat et al. 2012; Twum and Wei 2011; Wolff et al. 2008), assessing the prevalence of human exposure to these compounds is warranted.

Like many nonpersistent chemicals, DCPs or their precursors can be metabolized rapidly via phase I (e.g., oxidation) or phase II (i.e., conjugation) biotransformations and eliminated in urine. The urinary concentrations of total (free plus conjugated) species of DCPs have been used as exposure biomarkers (Hill et al. 1995; Hissink et al. 1997). Urinary concentrations of 2,4-DCP and 2,5-DCP in the general U.S. population have been measured as part of the National Health and Nutrition Examination Survey (NHANES), conducted by the Centers for Disease Control and Prevention (CDC 2012). In the present study, we examined data from NHANES 2003–2004, 2005–2006, 2007–2008, and 2009–2010 cycles (CDC 2012) to evaluate exposure trends during this 8-year period and to evaluate potential differences in urinary concentrations by race/ethnicity, age, sex, and income. Because housing-related environmental hazards may affect health (CDC 2011a), we therefore also examined housing type as a predictor of the urinary concentrations of DCPs.

## Materials and Methods

Urine samples analyzed for 2,4-DCP and 2,5-DCP were obtained from 10,426 participants ≥ 6 years of age from NHANES 2003–2010. The CDC’s National Center for Health Statistics Institutional Review Board reviewed and approved the NHANES study protocol. All participants gave informed written consent; parents or guardians provided consent for participants < 18 years of age (CDC 2006b). We quantified the urinary concentrations of 2,4-DCP and 2,5-DCP by online solid-phase extraction coupled to isotope dilution–high performance liquid chromatography–tandem mass spectrometry (Ye et al. 2005). The limit of detection (LOD) for 2,4-DCP was 0.2 μg/L for all four surveys; the LOD for 2,5-DCP was 0.1 μg/L for NHANES 2003–2004 and 0.2 μg/L (NHANES 2005–2010) (CDC 2012). Details of the analytical procedures used are available to the public on the NHANES website (CDC 2006a).

For statistical analyses, we used SAS (version 9.2; SAS Institute Inc., Cary, NC) and SUDAAN (version 10; Research Triangle Institute, Research Triangle Park, NC) programs. For each NHANES 2-year cycle, SUDAAN incorporates sample weights and design variables to account for the complex sample design of NHANES. We calculated the frequency of detection, the geometric means (GMs), and distribution percentiles for both the volume-based (in micrograms per liter) and creatinine-corrected (in micrograms per gram creatinine) concentrations. For concentrations below the LOD, as recommended for the analysis of NHANES data (CDC 2006a), we used a value equal to the LOD divided by the square root of 2 (Hornung and Reed 1990). Because DCP urinary concentrations were not normally distributed, we used the log_10_ transformation. Statistical significance was set at *p* < 0.05.

We used analysis of covariance to examine the relations of various demographic parameters and survey cycle to log_10_-transformed urinary concentrations of DCPs. Initial models included sex, age, race/ethnicity, family income, ln-transformed creatinine, and survey period, plus all possible two-way interactions. We categorized race/ethnicity on the basis of self-reported data as non-Hispanic black, non-Hispanic white, and Mexican American. We excluded 929 persons not defined by these racial groups in the analysis of covariance, but included these persons in the total population estimates. We stratified age, reported in years at the last birthday, into four groups (6–11, 12–19, 20–59, and > 60 years). In addition, we categorized family income based on the poverty income ratio (PIR, an index calculated by dividing family income by a poverty threshold specific to family size) (CDC 2011b) as below poverty (PIR < 1), low (PIR = 1–1.93), middle (PIR = 1.93–3.71), and high (PIR > 3.71). We estimated adjusted GMs using the regression equation with the intercept and regression coefficient for a given level of the categorical variable specified, and multiplied the β coefficient for all other categorical covariates by their estimated weighted percentage distribution.

To reach the final reduced models, we used backward elimination with a threshold of *p* < 0.05 for retaining covariates and two-way interactions, using Satterwaite-adjusted *F* statistics. In addition, we evaluated potential confounding by covariates that were not significant predictors by adding each back to a model that included significant predictors only. If addition of one of these excluded variables caused a change of ≥ 10% in the β coefficient for any of the significant predictors, we re-added the variable to the model. The final regression model included the following significant predictors of both DCPs (*p* < 0.01): age, survey period, and the interaction of race/ethnicity and family income.

Housing type information, collected from the NHANES housing characteristics questionnaire, was only available for the NHANES 2003–2004 and 2005–2006 cycles. We categorized the type of housing into four categories: apartment, single-unit house, mobile home/dormitory, and attached home. We calculated the distribution of housing type by race/ethnicity and family income categories. To evaluate the relationship between single-family housing and both income and race/ethnicity, we used weighted logistic regression, with housing type as the dependent variable [1, single-family house; 0, other type (i.e., multiunit) housing] and income level, race/ethnicity, and their interaction terms as independent variables.

To investigate the association between the likelihood of DCP concentrations being above the 95th percentile and type of housing, we first conducted weighted logistic regressions using type of housing (single-unit house, apartment, mobile home/dormitory, attached home) as the independent variable and a dichotomous variable to indicate whether the DCP concentration was above or below the 95th percentile as the dependent variable. Because the effect estimates for apartment, attached home, or mobile home/dormitory were similar (data not shown), we collapsed housing type into “single-unit house” and “multiunit house” and ran a second set of logistic regression models using the revised housing type categories adjusted by race/ethnicity (Mexican American, non-Hispanic white, non-Hispanic black, other) and income level.

We used the publicly available NHANES biomonitoring data on triclosan (CDC 2013) to determine weighted Pearson correlations between log_10_-transformed urinary concentrations of 2,4-DCP and triclosan for NHANES 2003–2010 participants. For a subset of NHANES 2003–2004 and 2005–2006 adult participants (20–59 years of age), we also determined weighted Pearson correlations among log_10_-transformed DCP concentrations and publicly available NHANES blood concentrations of 1,4-D (CDC 2013).

## Results

2,4-DCP (81.2–90.5%) and 2,5-DCP (97.4–98.3%) were frequently detected during the four survey periods examined, with GMs ranging from 0.803 μg/L to 1.04 μg/L for 2,4-DCP, and 6.1 μg/L to 12.9 μg/L for 2,5-DCP (Table 1). Distributions according to NHANES cycle, age, sex, and race/ethnicity are provided in Supplemental Material, Tables S1 and S2 (for 2,5-DCP and 2,4-DCP, respectively) and Tables S3 and S4 (for creatinine-corrected DCPs). The urinary concentrations of 2,5-DCP and 2,4-DCP among NHANES 2003–2010 participants (*n* = 10,426) were highly correlated (Pearson correlation coefficient (*r*) = 0.95, *p* < 0.0001); by contrast, the correlation between the urinary concentrations of 2,4-DCP and triclosan, another chlorinated chemical monitored in NHANES, was relatively low (*r* = 0.35, *p* < 0.0001). Furthermore, 1,4-D blood concentrations and urinary DCP concentrations were correlated among the 1,381 NHANES 2003–2004 and 2005–2006 adult participants with available data (2,5-DCP: *r* = 0.74; 2,4-DCP: *r* = 0.69, both *p* < 0.0001).

**Table 1:** GMs and selected percentiles (95% CIs) of urinary concentrations (μg/L), and detection frequency of 2,4-DCP and 2,5-DCP in the U.S. population ≥ 6 years of age, NHANES 2003–2010.

Variable	NHANES 2003–2004	NHANES 2005–2006	NHANES 2007–2008	NHANES 2009–2010
2,5-DCP
GM	12.90 (10.10, 16.30)	9.55 (6.67, 13.70)	9.04 (7.22, 11.30)	6.10 (4.94, 7.53)
50th percentile	10.50 (8.00, 14.20)	8.10 (5.60, 11.50)	6.60 (5.50, 8.30)	4.70 (3.70, 5.90)
95th percentile	705 (342,1,330)	332 (175, 794)	473 (296, 753)	301 (168, 618)
Frequency of detection (%)	98.2	98.2	98.3	97.4
2,4-DCP
GM	1.04 (0.90, 1.21)	0.95 (0.79, 1.13)	0.97 (0.85, 1.11)	0.80 (0.73, 0.89)
50th percentile	0.90 (0.80, 1.10)	0.80 (0.70, 1.00)	0.80 (0.70, 0.90)	0.70 (0.70, 0.80)
95th percentile	21.30 (14.10, 29.50)	11.9 (7.00, 20.40)	12.60 (9.00, 18.10)	8.80 (6.40, 15.70)
Frequency of detection (%)	81.2	87.5	90.5	85.9
*n* = 2,525 for NHANES 2003–2004, 2,548 for 2005–2006, 2,604 for 2007–2008, and 2,749 for 2009–2010.				

**Figure 1:**
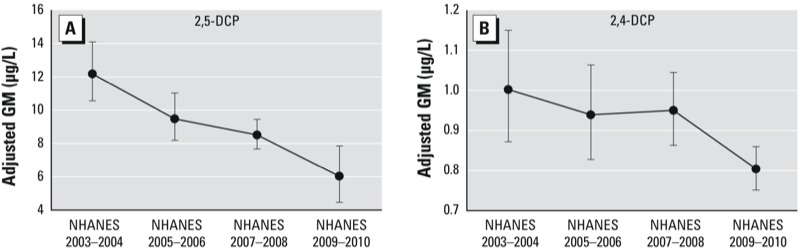
Temporal trend for adjusted GM urinary concentrations (μg/L) of 2,5-DCP (*A*) and 2,4-DCP (*B*). Adjusted GMs were estimated using the regression equation with the intercept and regression coefficient for a given level of the categorical variable specified, and multiplying the β coefficient for all other categorical covariates by their estimated weighted percentage distribution. Error bars represent 95% CIs.

Estimated GM concentrations of DCPs and corresponding 95% CIs (derived using weighted values for other model covariates) are listed in Table 2, and *p*-values for pair-wise comparisons between categories of each model predictor are provided in Supplemental Material, Table S5. Based on models adjusted for survey cycle and race/ethnicity × family income, GM concentrations of both 2,4-DCP and 2,5-DCP were significantly higher among 6- to 11-year-old children and older adults (≥ 60 years of age) than among 12- to 19- or 20- to 59-year-old participants (all *p* < 0.01). Adjusted GM concentrations of 2,5-DCP declined from 12.27 μg/L (NHANES 2003–2004) to 6.07 μg/L (NHANES 2009–2010) (Table 2, Figure 1). 2,4-DCP concentrations also decreased over time, although the decline was not monotonic and not as pronounced.

**Table 2:** Adjusted GM concentrations (95% CIs) of 2,4-DCP and 2,5-DCP (μg/L) according to age, NHANES cycle, and race/ethnicity × family income, NHANES 2003–2010.

Variable	Sample size (*n*)	2,5-DCP adjusted GM (95% CI)	2,4-DCP adjusted GM (95% CI)
Age group (years)
6–11	1,474	10.57 (8.93, 12.52)	1.12 (1.08, 1.16)
12–19	2,245	7.79 (6.59, 9.19)	0.88 (0.84, 0.91)
20–59	4,480	8.32 (7.44, 9.31)	0.88 (0.85, 0.90)
> 60	2,227	10.91 (9.34, 12.74)	1.06 (1.02, 1.10)
NHANES cycle
2003–2004	2,525	12.27 (10.10, 14.9)	1.01 (0.94, 1.07)
2005–2006	2,548	9.5 (7.12, 12.69)	0.95 (0.90, 1.010)
2007–2008	2,604	8.71 (7.17, 10.58)	0.97 (0.92, 1.01)
2009–2010	2,749	6.07 (5.05, 7.30)	0.80 (0.77, 0.84)
Race/ethnicity by family income^*a*^
Mexican American: below poverty	748	31.95 (22.54, 45.30)	1.95 (1.86, 2.04)
Mexican American: low	695	23.02 (16.60, 31.91)	1.69 (1.61, 1.77)
Mexican American: middle	467	18.83 (12.87, 27.56)	1.40 (1.30, 1.50)
Mexican American: high	235	9.28 (6.12, 14.08)	0.95 (0.83, 1.07)
Non-Hispanic white: below poverty	607	7.98 (6.37, 10.00)	0.86 (0.80, 0.92)
Non-Hispanic white: low	935	6.71 (5.49, 8.20)	0.74 (0.69, 0.79)
Non-Hispanic white: middle	1,078	6.47 (5.38, 7.80)	0.80 (0.77, 0.84)
Non-Hispanic white: high	1,551	6.21 (5.46, 7.06)	0.79 (0.75, 0.82)
Non-Hispanic black: below poverty	653	26.02 (19.81, 34.16)	1.53 (1.44, 1.62)
Non-Hispanic black: low	612	31.56 (23.39, 42.57)	1.83 (1.74, 1.92)
Non-Hispanic black: middle	577	26.55 (20.82, 33.87)	1.69 (1.62, 1.76)
Non-Hispanic black: high	435	20.70 (15.95, 26.88)	1.27 (1.18, 1.36)
Adjusted GMs were estimated using the regression equation with the intercept and regression coefficient for a given level of the categorical variable specified, and multiplying the β coefficient for all other categorical covariates by their estimated weighted percentage distribution. ^***a***^Below poverty, PIR < 1; low, PIR = 1–1.93; middle, PIR = 1.93–3.71; high, PIR > 3.71.			

**Figure 2:**
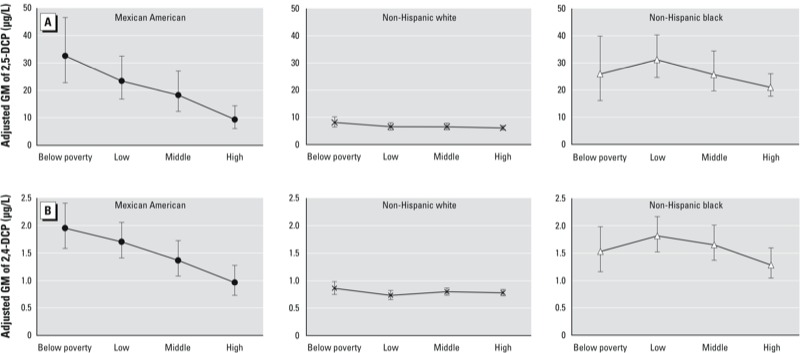
Adjusted GM urinary concentrations (μg/L) of 2,5-DCP (*A*) and 2,4-DCP (*B*) by family income categories. Below poverty, PIR < 1; low, PIR = 1–1.93; middle, PIR = 1.93–3.71; high, PIR > 3.71. Adjusted GMs were estimated using the regression equation with the intercept and regression coefficient for a given level of the categorical variable specified, and multiplying the β coefficient for all other categorical covariates by their estimated weighted percentage distribution. Error bars represent 95% CIs.

There was little variation in adjusted DCP concentrations among non-Hispanic whites according to income (Table 2, Figure 2; see also Supplemental Material, Table S5). Among Mexican Americans, DCPs decreased monotonically with increasing family income, from 31.95 μg/L (95% CI: 22.54, 45.3) for those below poverty to 9.28 μg/L (95% CI: 6.12, 14.08) in the highest-income group for 2,5-DCP, and from 1.95 μg/L (95% CI: 1.86, 2.04) to 0.95 μg/L (95% CI: 0.83, 1.07) for 2,4-DCP (both *p* < 0.001). DCP concentrations among non-Hispanic blacks also were lowest among those with the highest incomes, but the highest concentrations were in the low-income group (Figure 2). DCP concentrations were consistently lower in non-Hispanic whites than in Mexican Americans or non-Hispanic blacks, regardless of income, although the differences were not as pronounced among the high-income participants and no longer statistically significant for Mexican Americans and non-Hispanic whites. Concentrations of both DCPs were higher among Mexican Americans than non-Hispanic blacks among those below the poverty line, but higher among non-Hispanic blacks than Mexican Americans in all other income groups. Non-Hispanic whites lived in single-unit houses more often (75.2%) than Mexican Americans (60.5%) or non-Hispanic blacks (52.5%) (see Supplemental Material, Table S6). The percentage of people living in apartments or attached family housing decreased as family income increased, from 51.3% to 28.8% for non-Hispanic blacks, and from 32.8% to 12.4% for Mexican Americans (see Supplemental Material, Table S7). Among those with high incomes, fewer Mexican Americans (12.4%) than non-Hispanic blacks (28.8%) reported living in apartments or attached family houses.

Compared with non-Hispanic blacks, non-Hispanic whites and Mexican Americans were 1.95 times (95% CI: 1.44, 2.66) and 1.66 times (95% CI: 1.01, 2.74) more likely to live in single-family houses, respectively, based on logistic regression models adjusted for income. Compared with participants living below the poverty level, the odds of living in a single-family house increased with income [odds ratios (ORs) (95% CIs) were 1.62 (1.12, 2.35); 3.26 (2.18, 4.89); and 5.27 (3.5, 7.92) for low-, middle-, and high-income categories, respectively]. However, associations between living in a single-family home and race/ethnicity did not differ significantly according to income, and vice versa (data not shown).

The odds of having urinary DCP concentrations above the 95th percentile in NHANES 2003–2004 and 2005–2006 were significantly associated with type of housing (single vs. multiunit) and race/ethnicity, but not with income. After adjusting for race/ethnicity, participants living in multiunit housing were about 1.5 times more likely than participants living in single-family houses to have urinary concentrations of both DCPs above the 95th percentile (Table 3). Compared with non-Hispanic whites, Mexican Americans, non-Hispanic blacks, and persons of other races were 1.5–4.7 and 2.0–6.0 times more likely to have 2,4-DCP and 2,5-DCP concentrations above the 95th percentile, respectively (Table 3).

**Table 3:** Adjusted ORs (95% CIs) of the likelihood of participants having 2,4-DCP and 2,5-DCP urinary concentrations above the 95th percentile (NHANES 2003–2004 and NHANES 2005–2006).

Variable	2,5-DCP	2,4-DCP
Type of housing
Multiunit house^*a*^	1.48 (1.13, 1.93)	1.49 (1.16, 1.91)
Single-unit house (reference)	1.00	1.00
Race/ethnicity
Mexican American	6.05 (3.38, 10.82)	4.73 (2.65, 8.41)
Non-Hispanic black	5.80 (3.36, 10.00)	4.30 (2.69, 6.89)
Other	2.04 (0.95, 4.37)	1.53 (0.68, 3.43)
Non-Hispanic white (reference)	1.00	1.00
^***a***^Multiunit house includes apartments, attached homes, and mobile homes or dormitories.		

## Discussion

The detection of 2,4-DCP and 2,5-DCP in at least 81% of the samples from the four NHANES cycles examined suggests that exposure to these compounds or their precursors was widespread among the general U.S. population during 2003–2010. Depending on the survey cycle, the GM concentration of 2,5-DCP was 6–10 times greater than that of 2,4-DCP. Animal studies suggest that both 1,4-D and 2,4-D (precursors of 2,5-DCP and 2,4-DCP, respectively) are metabolized within hours and, thus, have short half-lives (HSDB 2014). Therefore, the differences between the GM urinary concentrations of 2,4-DCP and 2,5-DCP are likely related to different applications or production volumes of these two chemicals and their precursors. For example, in the United States the production volume of 2,4-DCP is lower than that of 2,5-DCP (HSDB 2014; NTP 2011; U.S. EPA 2002).

Degradation of triclosan in chlorinated water may result in 2,4-DCP formation (Canosa et al. 2005). However, the relatively low correlation between the urinary concentrations of 2,4-DCP and triclosan (*r* = 0.35) suggests that triclosan is not the main source of exposure to 2,4-DCP in these NHANES participants. The high correlation between 1,4-D concentrations in blood and urinary concentrations of 2,5-DCP (*r* = 0.74) supports the likelihood that consumer products containing 1,4-D are an important source of exposure and also supports the use of 2,5-DCP as a biomarker for exposure to 1,4-D, not only in occupational settings (Hsiao et al. 2009) but also among the general population. High correlations between urinary concentrations of 2,5-DCP and 2,4-DCP (*r* = 0.95) and between urinary DCPs and blood 1,4-D concentrations among NHANES participants suggest that people might be exposed to 2,4-DCP and 2,5-DCP through a common source (Lores et al. 1981).

Regardless of survey cycle, the adjusted GM of DCPs among children and older adults were significantly higher than for the other age groups. Although the reason for this finding is not clear, differences in lifestyles and/or metabolism of children and older adults may play a role (U.S. EPA 2008).

Adjusted GM concentrations of 2,5-DCP and 2,4-DCP both decreased between the 2003–2004 and 2009–2010 NHANES cycles based on models adjusted for age and race/ethnicity × income. Although 2,4-DCP, 2,5-DCP, and 1,4-D are all high-production volume chemicals, the production volume of 1,4-D has decreased since 2002 (U.S. EPA 2002). According to reports filed under the U.S. EPA’s Toxic Substances Control Act Inventory Update Rule, U.S. production plus imports of 1,4-D totaled 50–100 million pounds between 1990 and 2002, but decreased to 10–50 million pounds in 2006 (U.S. EPA 2002; NTP 2011). This downward trend in the use of 1,4-D may have contributed to the 50.5% decrease in the adjusted GM of 2,5-DCP from 2003–2004 to 2009–2010. For 2,4-DCP, for the same time period, the downward trend of adjusted GM of 2,4-DCP (19.8%) was not as pronounced as for 2,5-DCP.

We found that housing type was significantly associated with the likelihood of having urinary DCP concentrations above the 95th percentile. Specifically, we observed that, after adjusting for race/ethnicity, participants living in multiunit houses (i.e., apartments, attached homes, mobile homes/dormitories) were about 1.5 times more likely than participants living in single-family houses to have urinary DCP concentrations above the 95th percentile. Indoor air quality in a single-unit house is most likely unique to the home. By contrast, in apartments or in other types of attached housing, multiple dwellings may share, at least to a certain extent, the air quality because air may circulate among the closely connected living spaces. For example, evidence exists of secondhand smoke exposure in multiunit housing (King et al. 2010; Van Deusen et al. 2009). Our findings suggest that indoor air may be an important source of exposure to DCPs or their precursors (e.g., 1,4-D), particularly for people who live in close proximity to one another in multiunit housing.

Interestingly, Mexican Americans, non-Hispanic blacks, and persons of other races were 1.5–4.7 times more likely to have urinary 2,4-DCP concentrations above the 95th percentile, and 2.0–6.1 times more likely to have 2,5-DCP concentrations above the 95th percentile, than non-Hispanic whites. Because exposure to DCPs and their metabolites can occur through air inhalation, dermal contact, and ingestion of food and drinking water (HSDB 2014), associations with type of housing and race/ethnicity may be confounded by socioeconomic factors. For example, people living in multiunit housing might be more likely to consume food or drinking water contaminated with DCPs or their precursors than people living in single unit households. Also, people living in single-unit households in rural areas (e.g., farms) or in suburban settings may experience different exposures to DCPs from usage of pesticides, insecticides, or disinfectants. Unfortunately, because NHANES participants’ housing location information is confidential, we cannot differentiate suburban or rural settings from the available data. Nonetheless, because the U.S. population living on farms today is rather limited (16% in 2011; U.S. Census Bureau 2013), our findings should be generalizable to the general U.S. population, which for the most part does not live on farms.

Furthermore, adjusted GM concentrations of DCPs among non-Hispanic whites were consistently lower than among non-Hispanic blacks and Mexican Americans, although these differences, particularly for non-Hispanic whites and Mexican Americans, were not as pronounced among participants in high-income households. On the other hand, adjusted GMs of DCPs among non-Hispanic blacks and Mexican Americans were lowest among high-income participants relative to other income groups, with a monotonic decrease with income among Mexican Americans. Regardless of household income, non-Hispanic whites lived in single-unit houses more often than persons of other race/ethnicities. However, the percentage of non-Hispanic blacks and Mexican Americans living in apartments or in attached family housing decreased considerably when family income increased. In addition, in the high-income category, fewer Mexican Americans reported living in apartments or attached family houses than non-Hispanic blacks. Taking into consideration the above factors, we speculate that as household income increases, living in single-unit houses rather than in multiunit dwellings may have contributed to the downward trend of adjusted GM of DCPs for non-Hispanic blacks and, most evident, for Mexican Americans. It is also possible that low–household-income minority participants use room deodorizers and moth repellents to a greater extent than other population groups do, and indoor air concentrations of 1,4-D could exceed outdoor concentrations by at least an order of magnitude when room deodorizers and moth-control products are used (NTP 2011).

## Conclusions

Data from NHANES 2003–2010 suggest widespread exposure of the general U.S. population to DCPs or their precursors. The downward trend of DCP concentrations, particularly for 2,5-DCP, since 2003 suggests that decreasing exposures of the general U.S. population to DCPs and their precursors is likely related to decreases in production volumes of these compounds. We also observed differences in the adjusted GM concentrations of DCPs by age and race/ethnicity and a downward trend of adjusted GM DCP concentrations for non-Hispanic blacks and Mexican Americans with increasing family income. Housing type was a significant predictor of DCP urinary concentrations above the 95th percentile, suggesting that indoor air might be a likely route of human exposure to DCPs or their precursors. However, some of the differences above might also be partially related to socioeconomic disparities. The widespread exposure of the general U.S. population to DCPs, the differences in exposure by age, race/ethnicity, housing type, and socioeconomic status and the potential adverse health effects from exposure warrant additional research.

## Supplemental Material

(295 KB) PDF
