# Supplementary material for: Urinary Concentrations of 2,4-Dichlorophenol and 2,5-Dichlorophenol in the U.S. Population (National Health and Nutrition Examination Survey, 2003–2010): Trends and Predictors
Source: Environ Health Perspect. 2014 Jan 22;122(4):351–5. doi: 10.1289/ehp.1306816 (PMC3984229; doi:10.1289/ehp.1306816)
Supplement: (295 KB) PDF [file ehp.1306816.s001.pdf]

Environmental Health Perspectives doi:10.1289/ehp.1306816

Supplemental Material

Urinary Concentrations of 2,4-Dichlorophenol and 2,5-Dichlorophenol in the U.S. Population (National Health and Nutrition Examination Survey, 2003–2010): Trends and Predictors

Xiaoyun Ye, Lee-Yang Wong, Xiaoliu Zhou, and Antonia M. Calafat

Table of Contents

Supplemental Material, Table S1. Urinary concentrations in µg/L of 2,5-DCP (95th CI) stratified by age, sex, and race/ethnicity for NHANES cycles 2003–2004, 2005–2006, 2007–2008, and 2009–2010 **p. 2**

Supplemental Material, Table S2. Urinary concentrations in µg/L of 2,4-DCP (95th CI) stratified by age, sex, and race/ethnicity for NHANES cycles 2003–2004, 2005–2006, 2007–2008, and 2009–2010. **p. 3**

Supplemental Material, Table S3. Creatinine corrected urinary concentrations in µg/g creatinine of 2,5-DCP (95th CI), stratified by age, sex, and race/ethnicity from NHANES cycles 2003–04, 2005–2006, 2007–2008, and 2009–2010 **p. 4**

Supplemental Material, Table S4. Creatinine corrected urinary concentrations in µg/g creatinine of 2,4-DCP (95th CI), stratified by age, sex, and race/ethnicity from NHANES cycles 2003–04, 2005–2006, 2007–2008, and 2009–2010 **p. 5**

Supplemental Material Table S5. Observed statistical significance P values for differences between adjusted geometric mean urinary concentrations of 2,4-DCP and 2,5-DCP for various demographic groups **p. 6**

Supplemental Material Table S6. Weighted distribution of the type of housing by race/ethnicity for NHANES 2003-2004 and 2005-2006 **p. 8**

Supplemental Material Table S7. Distribution of housing type by family income and race for NHANES 2003–2004 and 2005–2006 cycles **p. 9**

Supplemental Material, Table S1. Urinary concentrations in µg/L of 2,5-DCP (95th CI) stratified by age, sex, and race/ethnicity for NHANES cycles 2003–2004, 2005–2006, 2007–2008, and 2009–2010.

	Survey years	Geometric mean	50th Percentile	75th Percentile	90th Percentile	95th Percentile	Sample size
Total	03-04	**12.9** (10.1,16.3)	**10.5** (8.00,14.2)	**40.9** (29.8,54.7)	**190** (133,282)	**705** (342,1330)	2525
	05-06	**9.55** (6.67,13.7)	**8.10** (5.60,11.5)	**26.4** (19.0,36.6)	**111** (69.9,166)	**332** (175,794)	2548
	07-08	**9.04** (7.22,11.3)	**6.60** (5.50,8.30)	**25.7** (19.2,34.7)	**131** (90.2,222)	**473** (296,753)	2604
	09-10	**6.10** (4.94,7.53)	**4.70** (3.70,5.90)	**18.4** (13.3,26.0)	**101** (68.0,146)	**301** (168,618)	2749
**Age group**							
6-11 years	03-04	**12.5** (8.22,18.9)	**9.10** (5.60,17.4)	**42.1** (21.7,83.9)	**161** (111,626)	**928** (249,1640)	314
	05-06	**10.5** (8.29,13.4)	**7.80** (5.90,10.6)	**28.1** (17.8,40.2)	**104** (55.4,226)	**336** (189,785)	356
	07-08	**9.31** (6.20,14.0)	**6.50** (4.60,10.9)	**23.3** (12.2,45.6)	**151** (61.1,306)	**464** (222,934)	389
	09-10	**7.19** (4.36,11.8)	**4.80** (2.70,9.90)	**30.4** (12.4,50.7)	**146** (63.7,368)	**503** (103,4940)	415
12-19 years	03-04	**16.9** (11.1,26.0)	**11.5** (8.20,20.6)	**49.9** (26.8,94.0)	**233** (94.5,1060)	**1080** (287,3970)	722
	05-06	**11.9** (8.47,16.8)	**9.60** (6.40,16.7)	**36.0** (22.3,54.4)	**127** (89.9,160)	**459** (160,894)	702
	07-08	**11.3** (8.78,14.5)	**7.40** (6.20,9.10)	**30.1** (18.5,52.6)	**193** (66.7,448)	**611** (254,1560)	401
	09-10	**8.01** (5.53,11.6)	**4.80** (3.50,8.80)	**24.9** (19.1,42.7)	**191** (61.2,368)	**526** (243,1140)	420
20 years and older	03-04	**12.3** (9.97,15.3)	**10.4** (8.00,14.0)	**40.5** (30.1,49.2)	**181** (141,250)	**583** (316,924)	1489
	05-06	**9.12** (6.15,13.5)	**7.80** (5.20,11.5)	**24.9** (17.3,35.3)	**110** (62.9,183)	**327** (159,852)	1490
	07-08	**8.71** (6.83,11.1)	**6.60** (5.10,8.70)	**24.6** (18.8,34.5)	**124** (95.9,186)	**452** (286,672)	1814
	09-10	**5.75** (4.77,6.92)	**4.60** (3.70,5.70)	**16.9** (12.7,23.1)	**88.4** (66.4,117)	**266** (156,450)	1914
**Sex**							
Males	03-04	**14.9** (11.8,18.8)	**12.5** (9.00,16.5)	**40.5** (30.7,54.5)	**152** (120,259)	**631** (259,1950)	1231
	05-06	**12.0** (8.55,16.7)	**9.90** (7.80,13.8)	**29.0** (21.8,40.5)	**114** (71.7,200)	**396** (175,916)	1270
	07-08	**10.9** (8.86,13.4)	**8.30** (6.30,9.90)	**29.4** (23.2,39.6)	**139** (100,248)	**546** (311,727)	1294
	09-10	**7.09** (5.68,8.85)	**5.30** (4.40,6.70)	**21.9** (15.2,33.5)	**103** (63.3,191)	**311** (133,736)	1399
Females	03-04	**11.2** (8.51,14.7)	**8.50** (6.30,12.0)	**42.8** (26.0,64.2)	**212** (141,364)	**732** (371,1100)	1294
	05-06	**7.69** (5.17,11.4)	**5.90** (3.90,9.40)	**21.1** (14.3,35.7)	**110** (60.5,183)	**317** (141,794)	1278
	07-08	**7.57** (5.70,10.1)	**5.50** (4.40,7.30)	**20.6** (13.9,31.8)	**118** (57.4,268)	**442** (213,838)	1310
	09-10	**5.28** (4.17,6.69)	**3.90** (3.00,5.10)	**16.7** (12.1,22.1)	**99.5** (58.2,156)	**287** (158,591)	1350
**Race/ethnicity**							
Mexican Americans	03-04	**30.1** (19.2,47.2)	**23.7** (14.7,39.8)	**103** (57.4,156)	**841** (282,2040)	**2370** (2040,3710)	617
	05-06	**32.2** (22.2,46.7)	**23.0** (14.9,37.1)	**120** (69.6,228)	**867** (298,1320)	**1630** (916,3650)	637
	07-08	**22.2** (9.88,49.8)	**16.4** (5.40,60.7)	**104** (35.2,364)	**566** (313,1710)	**1920** (672,3460)	531
	09-10	**13.0** (5.80,29.1)	**10.3** (3.30,26.2)	**53.6** (19.4,199)	**361** (124,900)	**998** (247,3700)	566
Non-Hispanic blacks	03-04	**54.0** (35.9,81.2)	**43.9** (26.2,65.6)	**159** (97.0,338)	**817** (342,2330)	**2330** (887,3730)	636
	05-06	**43.9** (33.2,58.1)	**33.2** (25.3,47.6)	**161** (79.9,255)	**722** (360,1370)	**1700** (886,6440)	678
	07-08	**27.4** (21.3,35.2)	**18.5** (13.2,26.7)	**102** (61.9,147)	**682** (364,943)	**1490** (933,1870)	597
	09-10	**23.0** (13.0,40.8)	**17.3** (8.00,42.7)	**82.7** (38.6,168)	**443** (119,2180)	**1240** (273,4940)	516
Non-Hispanic whites	03-04	**8.94** (7.15,11.2)	**7.80** (6.30,9.40)	**25.9** (19.3,36.6)	**115** (61.8,171)	**255** (148,522)	1077
	05-06	**6.19** (4.18,9.17)	**5.90** (3.80,9.20)	**15.7** (11.4,21.9)	**43.7** (31.2,73.1)	**105** (62.2,166)	1038
	07-08	**6.24** (5.04,7.74)	**5.10** (4.20,6.20)	**15.4** (12.2,19.9)	**49.8** (37.0,90.2)	**142** (103,294)	1077
	09-10	**4.10** (3.22,5.21)	**3.30** (2.60,4.40)	**10.9** (8.10,15.5)	**45.5** (29.0,82.7)	**124** (79.3,215)	1206

Supplemental Material Table S2. Urinary concentrations in µg/L of 2,4-DCP (95th CI) stratified by age, sex, and race/ethnicity for NHANES cycles 2003–2004, 2005–2006, 2007–2008, and 2009–2010.

	Survey years	Geometric mean	50th Percentile	75th Percentile	90th Percentile	95th Percentile	Sample size
Total	03-04	**1.04** (.895,1.21)	**.900** (.800,1.10)	**2.70** (2.30,3.10)	**8.80** (6.60,11.9)	**21.3** (14.1,29.5)	2525
	05-06	**.945** (.791,1.13)	**.800** (.700,1.00)	**2.00** (1.60,2.40)	**4.90** (3.90,6.30)	**11.9** (7.00,20.4)	2548
	07-08	**.970** (.852,1.11)	**.800** (.700,.900)	**1.80** (1.50,2.30)	**5.10** (3.80,7.60)	**12.6** (9.00,18.1)	2604
	09-10	**.803** (.729,.885)	**.700** (.700,.800)	**1.50** (1.40,1.70)	**4.00** (3.30,5.00)	**8.80** (6.40,15.7)	2749
**Age group**							
6-11 years	03-04	**1.01** (.796,1.28)	**.800** (.600,1.20)	**2.30** (1.70,3.20)	**7.70** (3.80,20.1)	**23.5** (9.40,31.0)	314
	05-06	**1.01** (.879,1.15)	**.800** (.800,1.10)	**2.00** (1.60,2.30)	**4.90** (3.30,6.60)	**9.80** (6.30,17.6)	356
	07-08	**1.04** (.778,1.39)	**.900** (.700,1.20)	**1.80** (1.20,2.80)	**5.90** (2.90,10.1)	**11.4** (6.60,20.7)	389
	09-10	**.975** (.768,1.24)	**.700** (.600,.900)	**1.80** (1.40,2.30)	**5.00** (3.20,8.40)	**14.2** (4.40,90.9)	415
12-19 years	03-04	**1.27** (.971,1.67)	**1.10** (.800,1.50)	**3.40** (2.50,5.00)	**13.6** (6.10,25.5)	**31.5** (14.5,85.0)	722
	05-06	**1.18** (.997,1.39)	**1.00** (.900,1.20)	**2.50** (2.00,3.10)	**5.50** (4.00,8.30)	**13.9** (7.10,33.6)	702
	07-08	**1.19** (.989,1.44)	**1.10** (.800,1.40)	**2.60** (2.00,3.00)	**5.60** (3.10,10.8)	**11.6** (5.70,36.5)	401
	09-10	**.967** (.794,1.18)	**.800** (.700,.900)	**1.60** (1.40,2.70)	**5.80** (3.70,10.1)	**14.4** (7.10,24.8)	420
20 years and older	03-04	**1.01** (.874,1.17)	**.900** (.700,1.10)	**2.60** (2.20,3.00)	**8.50** (6.60,10.4)	**19.4** (12.2,27.0)	1489
	05-06	**.907** (.737,1.12)	**.800** (.600,1.00)	**2.00** (1.50,2.40)	**4.90** (3.70,6.40)	**11.1** (6.50,20.9)	1490
	07-08	**.932** (.820,1.06)	**.800** (.700,.900)	**1.70** (1.40,2.20)	**5.00** (3.80,7.60)	**13.2** (9.20,18.1)	1814
	09-10	**.764** (.699,.836)	**.700** (.600,.700)	**1.40** (1.30,1.60)	**3.60** (3.10,4.50)	**8.00** (5.60,13.9)	1914
**Sex**							
Males	03-04	**1.22** (1.02,1.45)	**1.10** (.800,1.50)	**3.00** (2.50,3.50)	**9.40** (6.80,13.9)	**22.7** (13.6,40.9)	1231
	05-06	**1.16** (.973,1.37)	**1.00** (.900,1.20)	**2.40** (2.00,2.80)	**5.50** (4.40,7.90)	**12.9** (7.30,25.3)	1270
	07-08	**1.06** (.943,1.19)	**.900** (.800,1.00)	**1.90** (1.60,2.20)	**5.40** (3.90,8.20)	**13.6** (10.1,18.1)	1294
	09-10	**.879** (.789,.979)	**.800** (.700,.800)	**1.60** (1.40,1.80)	**4.00** (3.20,5.70)	**10.4** (5.20,18.4)	1399
Females	03-04	**.896** (.754,1.07)	**.800** (.600,.900)	**2.30** (2.00,2.70)	**8.10** (5.70,11.1)	**19.8** (12.0,27.5)	1294
	05-06	**.779** (.637,.954)	**.700** (.500,.800)	**1.50** (1.30,2.10)	**4.30** (2.80,6.20)	**9.40** (5.40,19.6)	1278
	07-08	**.893** (.750,1.06)	**.700** (.600,.800)	**1.80** (1.20,2.50)	**4.70** (3.10,8.00)	**11.9** (7.60,18.6)	1310
	09-10	**.737** (.659,.824)	**.600** (.600,.700)	**1.40** (1.30,1.70)	**4.00** (3.00,5.50)	**7.80** (5.80,15.8)	1350
**Race/ethnicity**							
Mexican Americans	03-04	**1.94** (1.46,2.56)	**1.70** (1.20,2.10)	**4.50** (2.80,9.30)	**26.9** (12.7,52.1)	**66.0** (47.5,84.2)	617
	05-06	**1.97** (1.49,2.59)	**1.60** (1.20,2.10)	**5.00** (3.30,6.60)	**20.9** (8.80,39.7)	**46.5** (21.9,79.5)	637
	07-08	**1.59** (.969,2.60)	**1.20** (.600,2.60)	**4.20** (2.10,9.50)	**13.4** (7.90,29.6)	**38.0** (16.4,74.0)	531
	09-10	**1.25** (.860,1.81)	**.900** (.700,1.30)	**2.70** (1.60,4.30)	**11.3** (4.30,26.3)	**29.1** (7.60,76.3)	566
Non-Hispanic blacks	03-04	**2.42** (1.92,3.06)	**2.20** (1.70,2.70)	**7.40** (4.00,9.60)	**20.8** (11.2,38.3)	**49.2** (24.0,69.7)	636
	05-06	**2.45** (1.93,3.12)	**2.10** (1.70,2.40)	**5.20** (3.90,7.40)	**20.3** (10.6,36.9)	**42.6** (21.3,129)	678
	07-08	**1.73** (1.49,2.01)	**1.40** (1.10,1.60)	**3.70** (2.90,4.90)	**17.8** (9.70,25.8)	**37.7** (24.6,56.8)	597
	09-10	**1.54** (1.06,2.23)	**1.20** (.800,2.00)	**3.10** (2.10,4.80)	**12.4** (4.30,46.4)	**35.2** (7.80,107)	516
Non-Hispanic whites	03-04	**.837** (.698,1.00)	**.700** (.600,.900)	**2.10** (1.70,2.60)	**6.20** (4.00,8.80)	**13.4** (8.60,22.0)	1077
	05-06	**.734** (.610,.883)	**.700** (.500,.900)	**1.40** (1.20,1.80)	**3.10** (2.70,3.90)	**5.30** (4.30,7.90)	1038
	07-08	**.817** (.732,.911)	**.700** (.600,.800)	**1.50** (1.20,1.80)	**3.10** (2.60,4.40)	**6.40** (4.60,8.80)	1077
	09-10	**.651** (.594,.712)	**.600** (.500,.700)	**1.30** (1.10,1.40)	**2.80** (2.20,3.50)	**5.60** (3.80,7.40)	1206

Supplemental Material Table S3. Creatinine corrected urinary concentrations in µg/g creatinine of 2,5-DCP (95th CI), stratified by age, sex, and race/ethnicity from NHANES cycles 2003–04, 2005–2006, 2007–2008, and 2009–2010.

	Survey years	Geometric mean	50th Percentile	75th Percentile	90th Percentile	95th Percentile	Sample size
Total	03-04	**12.5** (10.1, 15.6)	**9.29** (7.25, 12.5)	**34.4** (26.8, 45.4)	**141** (100, 251)	**578** (313, 851)	2522
	05-06	**9.31** (6.70,12.9)	**7.32** (5.33,10.2)	**20.4** (14.8,30.5)	**89.3** (54.9,176)	**292** (176,640)	2548
	07-08	**9.12** (7.35,11.3)	**6.24** (5.00,7.77)	**24.2** (17.2,30.3)	**109** (70.8,175)	**409** (234,745)	2604
	09-10	**6.36** (5.07,7.99)	**4.12** (3.31,5.16)	**16.2** (11.7,22.8)	**80.2** (59.5,127)	**269** (144,505)	2749
**Age group**							
6-11 years	03-04	**15.2** (9.93,23.1)	**10.6** (5.87,26.7)	**44.7** (28.9,80.0)	**183** (95.3,617)	**830** (330,2150)	314
	05-06	**11.6** (8.90,15.1)	**8.00** (5.95,12.6)	**24.7** (16.8,37.8)	**129** (55.8,242)	**419** (151,709)	356
	07-08	**11.5** (7.95,16.5)	**7.70** (5.41,11.7)	**29.5** (19.6,50.9)	**131** (59.9,239)	**420** (170,1110)	389
	09-10	**9.36** (5.65,15.5)	**6.25** (4.09,9.60)	**33.9** (12.6,65.0)	**177** (66.1,496)	**536** (111,5950)	415
12-19 years	03-04	**12.7** (8.50,18.9)	**9.05** (6.17,13.3)	**34.8** (18.6,67.0)	**177** (67.0,516)	**549** (187,2120)	720
	05-06	**8.88** (6.34,12.4)	**6.91** (4.15,11.1)	**23.4** (17.7,30.0)	**78.0** (58.5,112)	**279** (112,659)	702
	07-08	**8.79** (6.81,11.4)	**5.56** (4.42,7.50)	**20.9** (13.8,34.9)	**130** (41.8,251)	**353** (158,799)	401
	09-10	**6.44** (4.40,9.42)	**4.05** (2.34,8.69)	**19.4** (11.6,37.7)	**121** (49.3,218)	**257** (119,1180)	420
20 years and older	03-04	**12.2** (10.1,14.8)	**9.13** (7.25,12.4)	**32.7** (26.7,42.9)	**140** (103,203)	**552** (283,838)	1488
	05-06	**9.15** (6.43,13.0)	**7.29** (5.29,10.0)	**19.6** (14.2,30.4)	**90.7** (48.5,197)	**274** (163,701)	1490
	07-08	**8.94** (7.07,11.3)	**6.15** (4.93,8.00)	**24.3** (16.6,30.6)	**101** (70.0,162)	**422** (234,729)	1814
	09-10	**6.09** (4.97,7.45)	**3.97** (3.27,4.78)	**14.7** (11.0,20.5)	**72.5** (56.6,97.2)	**261** (141,446)	1914
**Sex**							
Males	03-04	**12.2** (9.73,15.3)	**9.65** (7.23,12.7)	**32.7** (25.3,39.1)	**108** (79.0,183)	**358** (161,1080)	1230
	05-06	**9.60** (7.17,12.9)	**8.11** (5.95,10.5)	**20.5** (15.9,28.2)	**74.9** (50.5,141)	**249** (137,534)	1270
	07-08	**9.17** (7.63,11.0)	**6.24** (5.22,7.81)	**25.5** (19.7,32.4)	**110** (70.7,167)	**353** (234,572)	1294
	09-10	**6.36** (5.04,8.02)	**4.21** (3.36,4.97)	**16.7** (11.7,24.0)	**83.2** (53.7,141)	**280** (111,727)	1399
Females	03-04	**12.9** (9.91,16.8)	**8.95** (6.98,13.2)	**37.1** (26.7,56.9)	**209** (124,362)	**660** (408,940)	1292
	05-06	**9.04** (6.18,13.2)	**6.60** (4.56,10.4)	**20.4** (14.1,33.8)	**104** (55.8,199)	**309** (149,933)	1278
	07-08	**9.07** (6.91,11.9)	**6.08** (4.71,8.04)	**22.4** (14.7,30.6)	**107** (59.2,216)	**509** (185,908)	1310
	09-10	**6.37** (4.92,8.25)	**4.10** (3.09,5.69)	**15.6** (11.3,22.1)	**77.1** (53.3,148)	**267** (151,481)	1350
**Race/ethnicity**							
Mexican Americans	03-04	**27.3** (17.4,42.9)	**17.8** (9.86,36.3)	**79.8** (45.8,138)	**809** (196,2110)	**2200** (1250,2480)	616
	05-06	**29.0** (20.5,41.0)	**18.6** (12.9,27.3)	**99.4** (61.6,165)	**675** (296,1400)	**1680** (718,2720)	637
	07-08	**21.6** (9.44,49.6)	**16.4** (5.09,58.9)	**83.9** (30.8,281)	**572** (182,1490)	**1490** (700,2220)	531
	09-10	**12.9** (5.78,28.8)	**9.12** (3.38,26.7)	**45.0** (17.3,236)	**460** (105,1000)	**1000** (380,2800)	566
Non-Hispanic blacks	03-04	**37.1** (24.3,56.7)	**27.4** (17.5,47.7)	**103** (63.8,216)	**609** (248,1210)	**1240** (627,2430)	635
	05-06	**30.9** (23.6,40.3)	**20.5** (17.3,29.1)	**104** (57.7,180)	**480** (294,1080)	**1480** (515,3100)	678
	07-08	**21.3** (16.2,27.9)	**14.6** (10.2,19.7)	**64.6** (48.0,101)	**529** (217,884)	**1130** (793,1560)	597
	09-10	**16.7** (9.19,30.2)	**12.5** (6.74,22.0)	**58.9** (22.3,106)	**349** (92.6,878)	**878** (277,3890)	516
Non-Hispanic whites	03-04	**9.24** (7.48,11.4)	**7.14** (5.67,8.76)	**24.8** (18.7,31.7)	**79.7** (50.2,141)	**216** (124,516)	1076
	05-06	**6.52** (4.51,9.43)	**5.60** (3.86,8.51)	**14.1** (10.7,19.7)	**40.2** (28.4,61.6)	**110** (47.3,224)	1038
	07-08	**6.52** (5.26,8.07)	**4.89** (3.95,6.20)	**14.4** (11.4,19.2)	**53.2** (40.1,75.9)	**131** (82.5,249)	1077
	09-10	**4.60** (3.59,5.90)	**3.27** (2.77,4.10)	**10.4** (7.22,15.2)	**41.3** (25.0,69.1)	**130** (73.4,180)	1206

Supplemental Material Table S4. Creatinine corrected urinary concentrations in µg/g creatinine of 2,4-DCP (95th CI), stratified by age, sex, and race/ethnicity from NHANES cycles 2003–04, 2005–2006, 2007–2008, and 2009–2010.

	Survey years	Geometric mean	50th Percentile	75th Percentile	90th Percentile	95th Percentile	Sample size
Total	03-04	**1.02** (.873,1.18)	**.880** (.770,1.00)	**2.19** (1.84,2.73)	**7.39** (5.00,9.83)	**15.4** (11.1,20.9)	2522
	05-06	**.922** (.798,1.06)	**.750** (.660,.880)	**1.58** (1.33,1.86)	**4.00** (3.00,5.71)	**8.90** (5.98,16.6)	2548
	07-08	**.978** (.867,1.10)	**.790** (.700,.880)	**1.63** (1.36,1.89)	**4.00** (3.14,5.63)	**11.7** (6.82,18.9)	2604
	09-10	**.838** (.757,.929)	**.680** (.630,.740)	**1.33** (1.18,1.58)	**3.48** (2.77,4.55)	**8.17** (5.44,14.3)	2749
**Age group**							
6-11 years	03-04	**1.23** (.965,1.56)	**1.03** (.750,1.45)	**2.39** (1.82,3.36)	**9.29** (3.98,16.5)	**20.9** (12.9,38.1)	314
	05-06	**1.11** (.950,1.29)	**.970** (.800,1.08)	**1.74** (1.38,2.19)	**4.38** (3.33,7.80)	**10.9** (5.12,23.3)	356
	07-08	**1.28** (1.00,1.63)	**1.06** (.750,1.40)	**2.06** (1.44,3.21)	**4.49** (3.13,9.27)	**11.2** (5.70,24.4)	389
	09-10	**1.27** (.986,1.64)	**.930** (.820,1.20)	**2.03** (1.54,2.94)	**5.89** (3.50,11.0)	**15.9** (5.71,121)	415
12-19 years	03-04	**.954** (.725,1.26)	**.790** (.660,1.00)	**2.08** (1.44,3.75)	**8.02** (4.72,12.5)	**14.8** (8.02,40.0)	720
	05-06	**.878** (.765,1.01)	**.700** (.600,.800)	**1.65** (1.22,1.93)	**3.92** (2.90,4.82)	**8.28** (4.82,15.9)	702
	07-08	**.927** (.776,1.11)	**.790** (.640,1.00)	**1.51** (1.14,2.22)	**3.81** (2.38,5.92)	**10.3** (4.28,21.8)	401
	09-10	**.778** (.656,.921)	**.580** (.510,.690)	**1.18** (.970,1.40)	**3.38** (2.11,6.27)	**7.38** (3.39,19.4)	420
20 years and older	03-04	**1.00** (.863,1.16)	**.870** (.770,1.00)	**2.17** (1.80,2.69)	**7.16** (4.88,9.01)	**15.0** (10.6,20.8)	1488
	05-06	**.909** (.774,1.07)	**.740** (.650,.870)	**1.55** (1.25,1.89)	**4.00** (2.84,6.19)	**8.80** (5.71,16.8)	1490
	07-08	**.958** (.847,1.08)	**.770** (.670,.880)	**1.60** (1.32,1.85)	**3.98** (3.14,5.59)	**12.1** (8.15,18.9)	1814
	09-10	**.810** (.735,.892)	**.670** (.620,.730)	**1.27** (1.11,1.56)	**3.33** (2.65,4.23)	**7.64** (5.16,12.3)	1914
**Sex**							
Males	03-04	**.995** (.850,1.17)	**.900** (.730,1.06)	**2.23** (1.82,2.82)	**6.84** (4.54,9.01)	**13.7** (9.29,21.8)	1230
	05-06	**.927** (.814,1.06)	**.770** (.670,.880)	**1.60** (1.36,1.86)	**4.12** (3.08,5.45)	**8.90** (5.19,16.6)	1270
	07-08	**.891** (.808,.984)	**.720** (.660,.790)	**1.44** (1.29,1.67)	**4.00** (2.97,5.30)	**9.96** (6.82,13.4)	1294
	09-10	**.788** (.706,.879)	**.620** (.580,.660)	**1.25** (1.06,1.48)	**3.66** (2.52,5.47)	**7.69** (4.84,16.9)	1399
Females	03-04	**1.03** (.845,1.27)	**.870** (.770,1.00)	**2.17** (1.73,2.73)	**8.00** (4.57,12.1)	**17.2** (11.1,23.7)	1292
	05-06	**.916** (.770,1.09)	**.740** (.640,.880)	**1.56** (1.19,1.96)	**3.91** (2.66,6.50)	**8.93** (5.53,23.7)	1278
	07-08	**1.07** (.910,1.26)	**.850** (.720,1.00)	**1.75** (1.43,2.29)	**4.07** (3.13,7.65)	**14.4** (6.50,26.8)	1310
	09-10	**.890** (.789,1.00)	**.740** (.660,.850)	**1.41** (1.24,1.67)	**3.39** (2.76,4.52)	**8.79** (5.16,14.8)	1350
**Race/ethnicity**							
Mexican Americans	03-04	**1.76** (1.30,2.38)	**1.33** (1.04,1.74)	**3.85** (2.29,8.81)	**23.8** (10.6,51.6)	**71.4** (30.8,88.8)	616
	05-06	**1.77** (1.38,2.27)	**1.25** (.990,1.73)	**3.79** (2.70,5.35)	**16.6** (6.75,31.8)	**38.1** (23.8,55.3)	637
	07-08	**1.55** (.925,2.60)	**1.18** (.630,2.28)	**3.33** (1.93,6.50)	**14.2** (5.65,30.6)	**33.1** (16.6,60.0)	531
	09-10	**1.24** (.860,1.78)	**.910** (.630,1.28)	**2.03** (1.43,3.90)	**11.0** (3.54,26.4)	**26.4** (10.2,93.5)	566
Non-Hispanic blacks	03-04	**1.66** (1.28,2.16)	**1.47** (1.06,1.96)	**4.14** (2.46,7.31)	**14.9** (7.93,20.1)	**22.9** (16.7,45.0)	635
	05-06	**1.72** (1.39,2.14)	**1.32** (1.11,1.56)	**3.28** (2.33,5.35)	**14.9** (7.40,28.1)	**37.0** (15.0,83.4)	678
	07-08	**1.34** (1.14,1.59)	**.990** (.800,1.17)	**2.36** (1.85,3.12)	**13.1** (5.70,23.3)	**33.8** (22.7,41.1)	597
	09-10	**1.11** (.739,1.67)	**.890** (.630,1.19)	**2.07** (1.25,3.87)	**8.37** (2.99,22.0)	**22.0** (7.05,83.1)	516
Non-Hispanic whites	03-04	**.864** (.721,1.03)	**.780** (.690,.890)	**1.86** (1.54,2.23)	**5.08** (3.58,8.00)	**10.8** (6.84,18.2)	1076
	05-06	**.772** (.660,.904)	**.670** (.580,.790)	**1.25** (1.07,1.56)	**2.78** (2.11,3.52)	**4.82** (3.33,8.62)	1038
	07-08	**.853** (.765,.950)	**.730** (.660,.810)	**1.36** (1.14,1.67)	**2.97** (2.53,3.33)	**5.16** (3.84,9.38)	1077
	09-10	**.731** (.674,.793)	**.630** (.580,.690)	**1.13** (1.01,1.25)	**2.76** (2.29,3.30)	**5.16** (3.79,6.46)	1206

Supplemental Material Table S5. Observed statistical significance P values for differences between adjusted geometric mean urinary concentrations of 2,4-DCP and 2,5-DCP for various demographic groups[a].

Differences	2,5-DCP	2,4-DCP
**Survey period**		
2003–2004 vs 2005–2006	0.148	0.559
2003–2004 vs 2007–2008	0.015	0.640
2003–2004 vs 2009–2010	<0.001	0.006
2005–2006 vs 2007–2008	0.619	0.851
2005–2006 vs 2009–2010	0.011	0.027
2007–2008 vs 2009–2010	0.009	0.004
**Age group (years)**		
6–11 vs 12–19	<0.001	<0.001
6–11 vs 20–59	0.003	<0.001
6–11 vs 60+	0.738	0.423
12–19 vs 20–59	0.328	0.967
12–19 vs 60+	<0.001	0.001
20–59 vs 60+	<0.001	<0.001
**Family income[b] by race/ethnicity**		
Below poverty vs low, Mexican American	0.035	0.171
Below poverty vs middle, Mexican American	0.009	0.007
Below poverty vs high, Mexican American	<0.001	<0.001
Low vs middle, Mexican American	0.263	0.127
Low vs high, Mexican American	<0.001	<0.001
Middle vs high, Mexican American	0.001	0.011
Below poverty vs low, non-Hispanic white	0.175	0.043
Below poverty vs middle, non-Hispanic white	0.062	0.268
Below poverty vs high, non-Hispanic white	0.021	0.173
Low vs middle, non-Hispanic white	0.688	0.140
Low vs high, non-Hispanic white	0.412	0.299
Middle vs high, non-Hispanic white	0.626	0.665
Below poverty vs low, non-Hispanic black	0.287	0.199
Below poverty vs middle, non-Hispanic black	0.888	0.301
Below poverty vs high, non-Hispanic black	0.184	0.198
Low vs middle, non-Hispanic black	0.299	0.501
Low vs high, non-Hispanic black	0.023	0.013
Middle vs high, non-Hispanic black	0.118	0.021

Differences	2,5-DCP	2,4-DCP
**Race/ethnicity by family income**		
Mexican American vs non-Hispanic white, at below poverty	<0.001	<0.001
Mexican American vs non-Hispanic black, at below poverty	0.323	0.080
non-Hispanic white vs non-Hispanic black, at below poverty	<0.001	<0.001
Mexican American vs non-Hispanic white, at low income	<0.001	<0.001
Mexican American vs non-Hispanic black, at low income	0.144	0.588
non-Hispanic white vs non-Hispanic black, at low income	<0.001	<0.001
Mexican American vs non-Hispanic white, at middle income	<0.001	<0.001
Mexican American vs non-Hispanic black, at middle income	0.111	0.203
non-Hispanic white vs non-Hispanic black, at middle income	<0.001	<0.001
Mexican American vs non-Hispanic white, at high income	0.060	0.155
Mexican American vs non-Hispanic black, at high income	0.001	0.091
non-Hispanic white vs non-Hispanic black, at high income	<0.001	<0.001

[a]We used analysis of covariance to examine the relations of various demographic parameters and survey cycle to log 10 transformed urinary concentrations of DCPs. The final regression model included the following significant predictors of both DCPs ($P < 0.01$): age, survey period, and the interaction of race/ethnicity and family income. [b]Family income: Below poverty (poverty income ratio < 1), Low (poverty income ratio: 1-1.93), Middle (poverty income ratio: 1.93-3.71), High (poverty income ratio > 3.71).

Supplemental Material Table S6. Weighted distribution of the type of housing by race/ethnicity for NHANES 2003–2004 and 2005–2006.

**Race/ethnicity**	**Apartment**	**Attached family house**	**Mobile or dormitory**	**Single family house**
Total (%)	14.53	6.97	7.73	70.76
Mexican American (%)	24.67	6.43	8.37	60.52
non-Hispanic white (%)	11.05	5.77	7.96	75.22
non-Hispanic black (%)	27.25	14.33	5.95	52.47

Supplemental Material Table S7. Distribution of housing type by family income[a] and race for NHANES 2003–2004 and 2005–2006 cycles.

Race/ethnicity	Apartment	Attached family house	Mobile or dormitory	Single family house
**Below poverty**				
Total (%)	26.21	10.44	18.82	44.53
Mexican American (%)	26.88	5.96	13.82	53.34
non-Hispanic white (%)	22.04	8.52	27.07	42.37
non-Hispanic black (%)	33.25	18.03	8.18	40.54
**Low**				
Total (%)	22.88	8.23	10.89	58
Mexican American (%)	32.69	8.15	5.74	53.42
non-Hispanic white (%)	17.34	6.99	13.15	62.52
non-Hispanic black (%)	34.27	12.69	7.19	45.84
**Middle**				
Total (%)	11.57	7.07	6.86	74.5
Mexican American (%)	17.99	7.32	5.77	68.91
non-Hispanic white (%)	9.32	5.93	7.18	77.57
non-Hispanic black (%)	22.2	14.58	5.48	57.75
**High**				
Total (%)	8.67	5.14	3.14	83.05
Mexican American (%)	11.09	1.27	5.74	81.9
non-Hispanic white (%)	7.9	4.72	3.13	84.25
non-Hispanic black (%)	17.11	11.66	2.28	68.94

[a]Family income categories: Below poverty (poverty income ratio <1), Low (poverty income ratio: 1-1.93), Middle (poverty income ratio: 1.93-3.71), High (poverty income ratio >3.71).
